# Advances in research on the carrot, an important root vegetable in the Apiaceae family

**DOI:** 10.1038/s41438-019-0150-6

**Published:** 2019-06-01

**Authors:** Feng Que, Xi-Lin Hou, Guang-Long Wang, Zhi-Sheng Xu, Guo-Fei Tan, Tong Li, Ya-Hui Wang, Ahmed Khadr, Ai-Sheng Xiong

**Affiliations:** 1 State Key Laboratory of Crop Genetics and Germplasm Enhancement, Ministry of Agriculture and Rural Affairs, Key Laboratory of Biology and Germplasm Enhancement of Horticultural Crops in East China, College of Horticulture, Nanjing Agricultural University, 1 Weigang, 210095 Nanjing, China; 2 School of Life Science and Food Engineering, Huaiyin Institute of Technology, 223003 Huaian, China; 3 Faculty of Agriculture, Damanhour University, Damanhour, Egypt

**Keywords:** Plant breeding, Plant hormones, Plant genetics

## Abstract

Carrots (*Daucus carota* L.), among the most important root vegetables in the Apiaceae family, are cultivated worldwide. The storage root is widely utilized due to its richness in carotenoids, anthocyanins, dietary fiber, vitamins and other nutrients. Carrot extracts, which serve as sources of antioxidants, have important functions in preventing many diseases. The biosynthesis, metabolism, and medicinal properties of carotenoids in carrots have been widely studied. Research on hormone regulation in the growth and development of carrots has also been widely performed. Recently, with the development of high-throughput sequencing technology, many efficient tools have been adopted in carrot research. A large amount of sequence data has been produced and applied to improve carrot breeding. A genome editing system based on CRISPR/Cas9 was also constructed for carrot research. In this review, we will briefly summarize the origins, genetic breeding, resistance breeding, genome editing, omics research, hormone regulation, and nutritional composition of carrots. Perspectives about future research work on carrots are also briefly provided.

## Carrots: past and future

China accounts for nearly half of the total global production of carrots and advances in genetic technologies are contributing to improve both crop quality and yield. Cultivated carrot is the second most popular vegetable in the world after potato; this can be largely attributed to their taste and their nutritional benefits. Ai-Sheng Xiong and colleagues at Nanjing Agricultural University, China, review the latest studies on the origin and breeding of carrots. Understanding the genetic sequence and gene expression patterns in carrot plants provides valuable insights into their evolution as well as into the mechanisms underlying their resistance to disease, production of healthy carotenoids and environmental stress tolerance. Future application of gene editing technologies will help to further improve crop production and the nutritional value of carrots.

## Introduction

Carrot (*Daucus carota* L.), a biennial herbaceous species, is a member of the Apiaceae family^1^. The cultivated carrots are mainly classified into eastern carrots and western carrots based on pigmentation in the carrot roots^2^. Eastern carrots are thought to originate from Afghanistan, while the origin of western carrots is still uncertain^2,3^. The roots of most eastern carrots are purple, and some are yellow. They have slightly dissected leaves and branched roots. The roots of most western carrots are orange, red or white. The leaves of western carrots are highly dissected, and the roots are unbranched^2,4^. Currently, orange carrots are becoming more popular and more widely cultivated in the world.

The carrot storage root is a good source of carotenoids, vitamins, and dietary fiber and is also rich in minerals and antioxidants^5,6^. With increasing health awareness, carrots are becoming more popular due to their abundant nutrients and benefits for human health. The majority of studies on carrots have focused on cultivation, breeding, tissue culture, nutrient content, and carotenoid synthesis regulation^7–9^. With the development of molecular biology technology, a large amount of information has been produced in research on vegetable crops. As one of the most important members of the Apiaceae family that is widely cultivated around the world, a large number of studies on carrots have also been performed. This review is mainly focused on the domestication, breeding, omics, and chemical composition of carrots. Potential further work in carrot research is also discussed.

## Biology and origins

### Biology

**Fig. 1:**
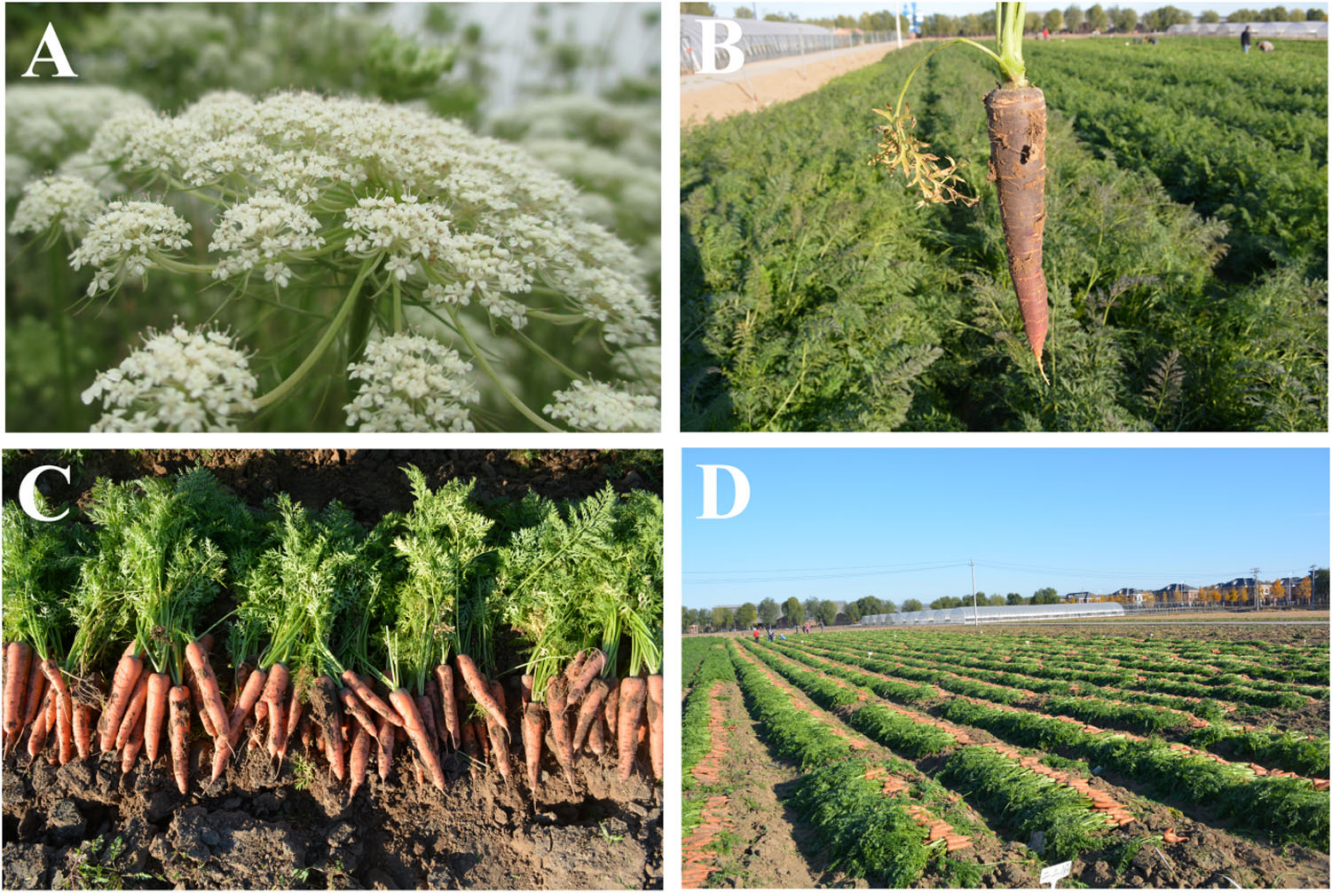
Pictures of carrot flowers, roots and field production. **a** Carrot flower. **b** Carrot root. **c**, **d** Field production of carrot

The carrot is a biennial herbaceous species in the Apiaceae family. Carrot roots, which develop from the hypocotyls, have good storage ability. A large amount of carbohydrates are stored in the enlarged taproots for the carrot plant flowering in the second year. The flower of the carrot is a flattened umbrella-shaped umbel (Fig. 1). The umbel is a characteristic for distinguishing carrots from related taxa. The colors of the cultivated carrot flowers are usually white, and the carrot leaves are compound leaves^4,10^. The fleshy taproot of the carrot develops from the hypocotyls, and the shape of the carrot root is always conical. The color of the root is varied and includes orange, yellow, purple, red, and white^4^. Different pigment contents are responsible for the different colors. With the further development of sequencing, more functional genes related to pigment synthesis will be found. The basic chromosome number of carrots is 9–11. Most cultivated carrots are diploid (2*n* = 2*x* = 18). The average length of the carrot chromosome is 2.34 μm^11,12^. In 2011, Iovene integrated the linkage groups with pachytene chromosomes of carrot through fluorescent in situ hybridization. In the report, the lengths of carrot chromosomes were only 2–4 μm, and the nine chromosomes were classified into three groups. Chromosomes 1, 6, and 8 with subterminal centromeres were a group; chromosomes 2 and 4 with terminal centromeres were a group; and chromosomes 3, 5, 7, and 9 with nearly median centromeres were a group. Among the nine chromosomes, chromosome 1 was the longest chromosome and had a small heterochromatic knob^13^.

Carrot is a cool climate crop that can be sown in spring in temperate climate zones or in the autumn or winter in subtropical climate zones^14^. Carrots are biennial plants. Vegetative growth is the main process of the first year of the life cycle to store material for reproductive growth. The flesh taproot collected for eating or selling is the root produced in the first year. Carrots will flower or bolt after vernalization when the roots are left in the ground. The time for vernalization must be at least 6 weeks. However, some wild carrots will flower or bolt with little or no vernalization^15,16^.

### Origin

The time frame and geographical location(s) of the earliest cultivated carrots are still uncertain. In Vavilov’s opinion, Asia Minor and the inner Asiatic regions were the origin centers of cultivated carrots. In addition, regions including Kazakhstan, Kyrgyzstan, Tajikistan, Turkmenistan, and Uzbekistan were the basic centers of cultivated carrots in Asia^17^. In the Stolarczyk and Janickan report, Afghanistan was thought to be the first center of carrot diversity, and Turkey was the second^10^. With the development of sequencing technology, many molecular markers have been used in research on plant evolution. In Orizzo and his group’s research, single nucleotide polymorphisms (SNPs) were adopted to analyze the structure and phylogeny of wild and cultivated carrots. A clear separation was found between wild and cultivated carrots. Based on historical documents and experimental results, central Asia was thought to be one origin of cultivated carrot^18^. All of the above research supports the idea that central Asia was an origin of cultivated carrots.

### Domestication

Shape, color and flavor, etc. were surmised as selection criteria in the domestication of carrot^4,19^. The cultivated carrot can be mainly classified into the anthocyanin, or eastern-type, carrot (e.g., yellow or purple) and the carotene, or western-type, carrot (e.g., yellow, orange, or red) based on the pigmentation in the roots^1,20,21^. Western carrots are always cylindrical or tapered cylindrical in shape and have less pubescent leaves, higher provitamin A carotenoid content, and higher sugar content than eastern carrots^1^^,^^22^. Eastern carrots always have thicker, shorter, narrow, conical roots, have pubescent leaves, flower early, and are poor in provitamin A carotenoid content^1^.

The viewpoint that the eastern-type cultivated carrot was domesticated from the wild carrots in the area around Afghanistan is generally agreed upon^2,4^. A recent study based on the transcriptome data analysis also supports the hypothesis that the eastern-type cultivated carrot originated in Western Asia^3^. However, there are still some different viewpoints on the origin of western-type cultivated carrots. The western-type cultivated carrot was thought to originate from eastern-type carrots directly, based on the earliest molecular study about carrot domestication^18^. In contrast, Heywood held the idea that western-type cultivated carrots did not originate directly from the eastern-type carrot^2^. He summarized the hypothesis that there was a secondary domestication event in the domestication of western-type cultivated carrot^2^. According to a recent study, western-type orange carrots may also originate from eastern carrots by introgression from wild carrots^3^. Areas around Afghanistan are generally agreed to be the geographic regions of the first cultivation of eastern-type carrots. To determine the geographic regions of the first cultivation of western-type carrots, more genetic sequencing studies are needed.

## Carrot breeding

### Male sterile breeding

Carrot is an allogamous plant. The stamens usually mature earlier than pistils in carrots^24,25^. The rate of natural hybrids in carrots is very high, but the value of seed production by natural hybrids is uncertain^26^. Male sterile lines have been used in the hybridization breeding of many crops and have made hybridization breeding easier for many plants that are difficult to breed using artificial emasculation^27^. In heterosis breeding, male sterile lines were also widely used. F_1_-hybrid breeding based on cytoplasmic male sterility is the main method of carrot breeding^28^.

In carrot, “brown anther” type and “petaloid” type are the two types of male sterility^29–32^. The stamens of the “brown anther” type are deformed and brown-colored. Morelock has identified this type in many cultivated and wild carrots^33^. The stamens of the “petaloid” type are transformed into a petal-like shape^34^. This type was found by Thompson^31^ and Mccollum^32^ in wild carrot. The anthesis of the “brown anther” type is blocked at a late stage of meiosis, and the stamens of the “petaloid” type produce no pollen. In carrot breeding, the “petaloid” type of male sterility is used more often. In 2018, Tan et al. found a wild petaloid male sterile carrot line (Wuye-BY). The male sterile carrot line has no swollen storage root, petal or anther. The advantage of the F_1_ hybrid is obvious^35^. In their subsequent experiments, they found that the sequence length of ATP synthase subunit 6 (atp6) was shorter in the male sterile line (Wuye-D) and longer in the fertile line (Wuye-L). The *atp6* gene is related to the fertility of plants. They thought that the longer *atp6* gene was associated with carrot fertility and that the short atp6 gene was associated with carrot male sterility^36^. In a study by Szklarczyk et al., the sequence of the carrot *atp9* gene (atp9-1) in petaloid carrots (Sp-cytoplasm) was shorter than the sequence of *atp9-3* in N-cytoplasmic plants. In Sp-cytoplasm carrot, the enhanced expression level of *atp9-1* was thought to be responsible for the enhanced ATP9 accumulation^37^. However, male sterility is a genocytoplasmic system, and the mutation in the *atp6* gene sequence is not enough to determine plant male sterility. More genetic evidence is needed to determine plant male sterility.

### Molecular breeding

#### Molecular marker breeding

Molecular markers are a new way to identify germplasm resources based on DNA and mRNA polymorphisms. They can be used in identifying core collections and examining the genetic relationship between parents in breeding research^38^. In genetic diversity analysis, molecular markers are also useful^39^. In basic research and breeding of carrots, molecular markers have been widely used.

In a study by Briard and colleagues, random amplified polymorphic DNA (RAPD) was found to perform better than morphological or isoenzymatic markers in the varietal identification of carrot^40^. In the characterization of genetic diversity in *Daucus* varieties, 26 accessions of *Daucus* were discriminated into five *Daucus* species and seven *D. carota* subspecies by RAPD and amplified fragment length polymorphism (AFLP)^41^. Lian et al. classified 34 carrot resources into 5 groups and 8 subgroups with 20 random primers by RAPD^42^. In the research of Grzebelus and colleagues, RAPD and AFLP were used to analyze the genetic diversity of carrots. Four inbred lines and eight F1 hybrids were identified through AFLP technology^43^. Six sequence-tagged site (STS) primer combinations were used to identify the carrot petaloid type of cytoplasmic male sterility (CMS). Five CMS lines were classified into two groups, and eight fertile carrots were classified into six groups^44^.

In breeding carrots for resistance to leaf blight, quantitative trait locus (QTL) mapping technology was used to identify QTLs in carrots with new genetic backgrounds. Eleven QTLs were found in the two carrots with new genetic backgrounds of resistance to *Alternaria dauci*^45^. The QTLs for the synthesis of α-carotenes, β-carotenes, carotene lycopene, and precursors have also been found^46^. Among the molecular markers, simple sequence repeat (SSR) markers are an important way to analyze genetic diversity. In the research of Baranski et al., 88 carrot accessions mainly from Europe, North America, and Asia were collected. Thirty SSR loci were fully characterized in these carrot accessions. As a result, the genetic diversity of the Western gene pool was lower than that of the Asian gene pool^47^.

In these studies, many molecular markers have been identified. Among these markers, some are disease resistance-related, some are agricultural trait-related and some reflect genetic diversity. All these markers will play important roles in carrot breeding with the carrot genome sequence database.

#### Transgenic breeding

Carrot is known as one of the pioneer species in the research of plant tissue culture^48^. The transformation protocols of carrots have also been established over decades. Many transformation methods for carrots have been established. Among the diverse techniques, *Agrobacterium*-based systems are the most common methods^48,49^. *Agrobacterium* includes *A. tumefaciens* and *A. rhizogenes*, and *A. tumefaciens* is the most common strain in *Agrobacterium*-based systems. The first carrot transformation based on *A. tumefaciens* was reported in 1987 ^50^. According to many optimized transformation protocols of carrot transgenesis systems, explant type, variety, and bacterial strain were found to be the main factors affecting the transformation frequency^48,51^. In carrots, roots, cotyledons, hypocotyls, and petioles can all be used as explants. In Pawlicki and his group’s study, the transformation frequency was higher when petioles were used as the explants^51,52^. The time of cocultivation was also important. Compared with a cocultivation time of 1 or 7 days, the transformation frequency at a cocultivation time of 2 or 3 days was higher^51^. Following the development of the carrot transformation system, the media was also changed from MS to B5 ^53^.

In the breeding research on carrots, the genetic engineering method was also adopted. The approach based on overexpressing functional genes was widely used. In the research of Wally et al., functional genes (*OsPOC1*, *OsPrx114*, and *AtNPR1*) from other species were overexpressed in carrots to enhance fungal and disease resistance. The transgenic lines overexpressing *OsPrx114* displayed high disease resistance compared with the control^54^. In addition to resistance breeding, genetic engineering was also used to accumulate some special components by overexpressing characteristic genes in carrots. In the research of Luchakivskaya et al., *HuINFα-2b* was overexpressed in carrots to enhance the accumulation of human interferon alpha-2b protein. The transgenic lines expressing *HuINFα-2b* were thought to be useful in curing various viral diseases^55^.

In recent years, the genome editing method based on the CRISPR/Cas9 system has developed rapidly. This genome editing system has been applied in many spaces, including humans, mice, and plants^56–58^. The CRISPR/Cas9 system is a fast and easy way to edit the genome. In 2018, this genome editing system was first employed in carrot research. Klimek-Chodacka et al. knocked out the *F3H* gene in carrots and validated the importance of this gene in the biosynthesis of anthocyanin^59^. Xu et al. used the CRISPR/Cas9 system to knock out *DcPDS* in orange carrots and the *DcMYB113-like* gene in purple carrots, and the editing efficiencies were 35.35% and 36.4%, respectively^60^. These results suggest that the CRISPR/Cas9 system will be an important and useful method for further research on gene function in carrots.

The transformation method of carrots has matured. Transgenic breeding of carrots has been widely applied in experimental studies, and many important functional genes have been determined by overexpression or genome editing. However, this method should be used cautiously in field production.

### Disease and pest resistance breeding

#### Aster yellows

Aster yellows is an insect-vectored carrot disease caused by a mycoplasma-like organism. This disease is one of the important diseases that limits the growth and yield of carrot^61,62^. Carrots infected by this typical disease will show stunting, yellowing, leaf bronzing, sterility and leaf-like petals^63^. Breeding carrot disease resistance to aster yellows has been implemented for many years. Gabelman and his collaborators selected breeding lines with high resistance to aster yellows through field evaluation and selection. In the breeding process, “Scarlet Nantes”, “Royal Chantenay”, and “Gold King” were found to have higher resistance to aster yellows and “Danvers 126”, “Py-60”, and “Spartan Bonus 80” were more susceptible^63^.

#### Fungal leaf blight

Fungal leaf blight is a kind of foliar disease in the cultivation of carrots. Fungal leaf blights are mainly caused by *A. dauci* (Kühn) and *Cercospora carotae* (Pass.) Solheim around the world^64^. *A. dauci* lesions always occur on older leaves, and *C. carotae* lesions always occur on new leaves in carrots^65–67^. Fungal leaf blights are found to cause yield loss by reducing leaf photosynthetic area and breaking carrot petioles. The *A. dauci* and *C. carotae* lesions can break carrot seedlings by girdling their petioles^68^. Breeding for leaf blight resistance has been performed for many years. The less-susceptible cultivars are found to have characteristics that delay the spread rate of pathogens. According to Gugino’s report, “Bolero”, “Carson”, “Calgary”, “Ithaca”, and “Fullback” were the cultivars that were less susceptible to *A. dauci*, and “Bolero”, “Carson”, and “Bergen” were the cultivars that were less susceptible to *C*. *carotae*^68^. In the research of Le Clerc et al., QTL mapping technology was adopted for the resistance breeding of leaf blight, and 11 QTLs related to resistance to *A. dauci* were found^45^.

#### Carrot fly

The carrot fly, *Psila rosae* F. (Diptera: Psilidae), is the most serious and widespread pest in carrot production^69^. Carrot leaves turn red, orange or rust- colored, and roots present rusty brown scars and tunnels when infected by carrot flies. Carrots infected by carrot flies are inedible and unmarketable^70^. Reports about carrot fly resistance breeding are available from more than 100 years ago^69^. From 1977 to 1978, Ellis and his group performed carrot fly resistance breeding at 12 different locations in England. A total of eight varieties with different resistances were cultivated, including “Clause’s Sytan Original”, “Gelbe Rheinische”, “Vertou LD”, “Clause’s Jaune Obtuse de Doubs”, “Royal Chantenay Elite (Rota) No.275”, “Long Chantenay”, “Danvers Half Long 126”, and “St. Valery”. Among the eight varieties, “Sytan”, cultivated in Nantes, showed the highest resistances to carrot fly^71^. To compare the difference between carrots with different resistances to carrot flies, “Sytan” (most resistant) and “Danvers” (least resistant) were chosen for comparison. The results of the comparative experiment suggested that carrots with high resistance to carrot flies reduce fly damage by delaying the development of larvae^70^. To overcome the effect of environmental factors in variety selection, Ellis and his group developed inbred carrots by using a single seed descent program. A total of nine carrot lines with moderate resistance to carrot fly were selected and seeded^72^. Many other reports about carrot fly resistance breeding have shown that “Sytan” is the variety with the highest resistance. The resistance of “Sytan” to carrot flies has been demonstrated in Canada, Germany, Ireland, New Zealand, and the UK^69,73–75^. During breeding progress, it was realized that varieties with high carrot fly resistance have lower levels of chlorogenic acid than varieties with low resistance^76^. The concentration of chlorogenic acid may be used as a selection criterion to select a variety with high resistance to carrot flies. However, reports about genetic research into the resistance to carrot flies in carrots are still scarce.

#### Root-knot nematodes

Root-knot nematodes (RKNs) are significant pests that are widely present in plants^77^. In many carrot-producing regions, RKNs are major pests that limit carrot production^78^. In cooler producing regions, *Meloidogyne hapla* is the most prevalent. In warmer producing areas, *Meloidogyne javanica* and *Meloidogyne incognita* are the predominant RKN species^79^. The characteristics of RKNs (present in soil and having a broad host range) and the limited use of nematicides lead to difficulty in controlling the pathogen^80^. To guarantee carrot quality, it is necessary to perform root-knot nematode resistance breeding in carrots. Huang et al. found that “Brasilia” and “Tropical” are resistant to *M. javanica*^81,82^. “Brasilia” is the most promising source of resistance to *M. javanica*^83,84^. Through the use of molecular markers (RPAD and QTL), a locus named Mj-1 that imparts resistance to *M. javanica* was found in the “Brasilia” cultivar^79,85^. The Mj-1 locus is mapped on chromosome 8 ^79^. In 2014, another locus that imparts resistance to *M. javanica* was mapped in the cultivar “PI652188” and named Mj-2. Mj-2 is also on chromosome 8 but does not map to the same locus as Mj-1 ^86^. The discovery of Mj-1 and Mj-2 is meaningful for the root-knot nematode resistance breeding of carrots.

## Omics research

### Genomics research

Over the past few decades, sequencing technology has developed rapidly, and more than 100 plant genomes have been sequenced^7^. As the most important vegetable in the Apiaceae family, the carrot genome was also sequenced. In 2014, a genomic database for the carrot was released. This database provides de novo assembled whole-genome sequences and classified transcription factor families of carrots, which is helpful for further research on carrots^87^. Two years later, a high-quality carrot genome (421.5 Mb) was released. In this study, the evolution of the carrot genome was analyzed, and two new whole-genome duplications (WGDs) were identified. The two WGD possibilities occurred ~43 and ~70 million years ago, respectively. Furthermore, *DCAR_032551* was hypothesized to play roles in regulating photomorphogenesis and root de-etiolation of carrots. Based on the whole-genome sequencing of carrots, 32,113 genes were predicted. Among the 32,113 genes, 10,530 genes unique to carrots were found^88^. In 2018, the genome sequence data for “Kurodagosun”, a major carrot cultivar in Japan and China, was also released^89^. All of these genome sequence data will significantly promote research on carrot evolution, carotenoid synthesis, and many other important projects in carrots. The genome in Iorizzo’s research (421.5 Mb) is obviously larger than that in Wang’s research (371.6 Mb), which suggests a difference between the sequencing quality in the two studies. The genome data in Iorizzo’s research have higher quality and were analyzed more deeply. The database published by Xu provides a tool for researchers to download gene sequences from Wang’s research. In future research, the two carrot genome datasets should be combined in carrot studies.

The plastid genome of carrots has also been sequenced. In 2006, the plastid genome of carrot was sequenced by Ruhlman et al. The length of the carrot plastid genome is 155,911 bp with 115 unique genes. The results from that research provided a valuable resource for phylogenetic analysis among different angiosperms. In the phylogenetic analysis, the bootstrap values between *Daucus* and *Panax* were all 100% in the Maximum parsimony (MP) tree and Maximum likelihood (ML) tree. The results strongly supported the sister relationship between *Daucus* and *Panax*^90^.

### Transcriptomic research

Transcriptomics is an approach to studying gene expression by measuring all mRNA transcripts in one cell or tissue. The transcriptome sequence dataset is widely used in analyzing gene expression, discovering gene functions, and developing molecular markers^91,92^. In carrot research, transcriptomics was also widely adopted. In 2011, to build a molecular resource for revealing new markers and novel genes, the carrot transcriptome was de novo assembled and characterized by Simon’s group. To our knowledge, this transcriptome is the first transcriptome of carrot. Based on transcriptome sequencing, 114 computationally polymorphic SSRs and 20,058 SNPs were identified. In addition, polymorphisms were found predominantly between inbred lines^93^. Xiong’s group established the transcriptomic database (CarrotDB) in 2014. The database was established based on transcriptomic sequences from 14 carrot genotypes^87^.

To further investigate the domestication of carrots, the root transcriptomes of six cultivated carrots and five wild carrots were sequenced. Rong et al. thought that some other wild *D. carota* subspecies should also be involved in the research of carrot domestication. In cultivated carrots, the expression of the water-channel-protein gene and carotenoid-binding-protein gene was upregulated, and the expression of allergen-protein-like genes was silenced^3^. All these results revealed the potential role of regulators of gene expression in domestication. The western carrots were thought to originate from eastern carrots based on the analysis of transcriptome data from different cultivated and wild carrots.

Phytohormones play important roles in controlling plant root growth and development. As a root vegetable, the effect of hormones on carrot root growth should be investigated. To investigate the molecular mechanisms of hormones on carrot root growth, the transcriptomes of four different developmental stages were sequenced. A total of 4818 unigenes with differential expression between the four stages were identified. Among the 4818 unigenes, 87 genes were found to be involved in the hormone-related pathway ^94^. The transcriptome analysis of the key genes involved in the biosynthesis and signaling pathway of phytohormones helped to clarify the roles of hormones during root development.

To investigate the biosynthesis of carotenoids in carrot leaves and roots, Ma et al. sequenced the transcriptome of carrot leaves and roots. Based on the transcriptome data, DcPSY1 was thought to be the crucial factor responsible for the higher carotenoid content in carrot leaves. *DcLBCY*, *DcLECY*, and *DcZEP1* may be responsible for the differences in carotene and xanthophyll levels between carrot leaves and roots^95^.

Depending on the demands of different studies, various transcriptome sequence datasets have been generated. All the datasets are the sequences of genes that are expressed under certain conditions. Transcriptomics promotes research on carrots, and many single important genes were found through this method. On the other hand, research on the relationship among different genes is also interesting. In the future, more research on the interaction among genes based on transcriptome sequence datasets should be performed in carrots.

### microRNA research

microRNAs (miRNAs) are a type of noncoding small RNA ~20–24 nucleotides in length^96^. Numerous studies have found that plant miRNAs preferentially target transcription factors and play important roles in regulating plant development^97^. miRNAs are also important participants in responses to abiotic and biotic stresses ^98^. As a consensus, miRNA may be an important research object in research on improving the agronomic characteristics of crops.

The first carrot microRNA database was reported in 2013. Seventeen microRNAs were identified from the research. The 17 microRNAs came from 12 different families (dca-mir-156, 160, 167, 172, 774, 778, 854, 1310, 5015, 5030, 5658 and 5664). In analyzing the potential targets of the 17 microRNAs, 24 targets were determined. Among the 24 potential targets, 8 were transcription factors, 6 were stress related, 5 were involved in metabolism and 4 were related to plant growth. Most targets identified in the research have also been reported as microRNA targets in other plants^99^. The microRNA database and the findings are valuable resources for further research in improving the agronomic characteristics of carrots. Unfortunately, research about the microRNA in carrots is still scarce.

### Proteomic research

Proteomics is an important technology for studying the growth, development, and stress responses of plants by systematically analyzing the plant proteome. This technology is an important complement to the genome^100,101^. Through proteomics, numerous proteins involved in the complex signaling and metabolic network of plants can be qualitatively and quantitatively analyzed^100^. In stress-response research in plants, proteomics has proven to be a powerful method and has been used in studying drought, flooding, and nutritional stress responses^102^. Furthermore, proteomics has also been widely used in studying the mechanisms and biological processes of plants, such as fruit ripening, seed germination, and floral development^103^.

In the research of Louarn et al., proteomics was used to analyze changes in the proteome of carrots during cold storage. Carrots cultivated in two different cropping systems were selected as the research object. A total of 15 proteins were found to change in levels in the first month of storage. Between the two different cropping systems, the change in protein level was small. Among the 15 proteins, three were related to the stress response, and three were related to the cytoskeleton. The research indicated that carrot roots have an adaptation to the low temperature within the first month of cold storage^104^. In the research of Wang et al., isobaric tags for relative and absolute quantification (iTRAQ) were used to investigate the impact of elevated carbon dioxide (CO_2_) on carrot growth and development. Through proteome sequencing, they found a potential molecular mechanism for the altered lignin content in carrot roots induced by elevated CO_2_^105^. Proteomic technology provides a powerful way to understand the complex signaling and metabolic network of plant physiological progress and can contribute to further investigation of improving carrot yield and nutrition.

### Metabolomic research

Metabolomics is a kind of functional genomics that measures all small molecules (molecular masses ≤ 1500 Da) in a cell or a tissue. The cell or the tissue measured is always in a particular physiological or developmental state^106^. This is a novel approach to performing qualitative and quantitative studies on plant biochemistry at a global level. Metabolomics has been widely adopted to study plant metabolism and food quality^107^.

To investigate the difference between wild and cultivated carrots, Grebenstein et al. constructed the metabolic fingerprinting of wild (“Dutch”) and cultivated (western orange) carrots through nuclear magnetic resonance spectroscopy (NMR). Differences between the two kinds of carrots only appeared at the quantitative level in the metabolic content. The metabolome of the hybrid of wild and cultivated carrots showed high similarity to that of the maternal carrot. The maternal characteristics and maternal environment may be the factors leading to the similar metabolic content between hybrid and maternal carrots^108^.

In carrot breeding research, metabolomics is also used as a powerful tool. In Leiss and his group’s research, metabolomics was adopted to investigate the resistance of different carrot varieties to Western flower thrips. A cultivated carrot (Ingot) was found to have the highest resistance. The wild and biofortified carrots did not show distinct resistance to the thrips. The contents of the flavanoid luteolin, the phenylpropanoid sinapic acid and the amino acid balanine were more abundant in the resistant carrots. These results were useful in the research of thrip resistance breeding in carrots^109^.

In 2014, the metabolomes of five carrot varieties were measured by NMR to study the effect of genotype on the metabolite components of carrot. In this study, orange carrot varieties were found to be more abundant in the contents of sucrose and β-carotene and to be lacking in the contents of fructose and glucose. The difference between yellow and white carrot varieties was unclear. Genetic differences, growing strategies, and soil types were finally determined to be the factors that affect the composition of different carrot varieties. The results are useful in breeding to improve carrot quality^110^.

## Hormone regulation

Phytohormones are important regulators of plant growth and environmental responses. They are involved in almost all physiological processes during the growth and development of plants, such as cell division, growth and differentiation, flowering, seed development, and senescence^111,112^. Cytokinins (CK), abscisic acid (ABA), auxins (Aux), ethylene (ET), gibberellins (GA), brassinosteroids (BR) and jasmonates (JA) are the most studied hormones in plants^111^. Among these hormones, Aux, GA, BR and CK are mainly related to plant development; JA and ET are mainly related to plant defense; and ABA is related to the abiotic stress response^111,113^. Research on the roles of ABA, GA, BR, CK, Aux, and JA in growth and development of carrots has been performed.

### Auxins

Auxin is a pivotal plant hormone whose cellular level is important for regulating plant growth and development. Fruit formation, leaf abscission, cell division, and cell elongation were all reported to be regulated by auxin^114,115^. Among the different kinds of naturally active auxins, IAA is the best studied^116^. In the research of Wu et al., contents of IAA changed obviously at different growth stages and showed distinct discrepancies in different organs. In addition, 18 genes involved in the biosynthesis and signaling pathway of IAA were identified. The way that IAA regulates carrot growth and development may be tissue-specific^117^. However, there are few reports on the effect of exogenous IAA on the field production of carrots.

### Gibberellins

Gibberellins are diterpenoid compounds. Through the whole life cycle of plants, GAs play pivotal roles in regulating growth and development, including seed germination, stem elongation, flowering, and fruit development^118^. In cellular growth, promoting the elongation and expansion of cells are the main functions of GAs. Mutants that are defective in GA biosynthesis are always dwarfs^119^.

During the growth and development of carrots, GAs play pivotal roles. In carrots, the content levels in roots are lower than those in petioles and leaf blades^120^. Through foliar application, exogenous GA_3_ promotes the growth of the aboveground part and inhibits the growth of roots^119^. At higher temperatures, GA spray application can influence carrot flowering^121^. The impact of GAs on carrot roots promotes the development of secondary xylem and decreases the proportion of secondary phloem^122^. In a study by Wang et al., exogenous GA_3_ was shown to enhance the lignification of carrot roots^123^. In addition, GA was involved in the regulation of the differentiation of embryogenic cells in carrot^124^.

### Brassinosteroids

Brassinosteroids (BRs) are steroid hormones, an important kind of plant regulator^125^. To date, more than 70 brassinosteroid compounds have been isolated, and brassinolide is the most bioactive compound^126^. During plant growth and development, BRs are involved in various biological processes, such as the formation of stomata and lateral roots, flowering, and fruit maturation. BRs also play important roles in promoting cell division, vascular differentiation and cell elongation, and enhancing the tolerance of the plant^127,128^. The spatiotemporal distribution of brassinosteroid activity is a decisive factor that influences the function of BRs^129^. However, the precise spatial and subcellular distribution of BRs in plant organs is still unclear. Regarding the biosynthesis and signal transduction of BRs, the related genes have been determined by using BR-related mutants in *Arabidopsis* and many other plants^125^. In carrots, genes involved in the biosynthesis and signal transduction pathways have been identified via transcriptomic research. Foliar application of 24-epibrassinolide was proven to promote the elongation of carrot petioles. The aboveground part of the carrot treated with 24-epibrassinolide was taller and heavier than that without treatment^130^.

### Abscisic acid

Abscisic acid (ABA) was first recognized as a plant hormone in the early 1960s^131^. ABA plays important roles in regulating almost all physiological processes during plant growth and development, including seed dormancy, seed germination, and fruit maturity^132^. In the presence of environmental stress, ABA plays important roles in inducing stomatal closure to respond to water deficiency^133^. The concentration of ABA within plants determines the roles of ABA in response to changing physiology. In carrots, most reports about ABA are related to somatic embryogenesis. Kiyosue et al. measured the concentration of endogenous ABA in the embryogenic cells, nonembryogenic cells and somatic embryos of carrot. They found that embryogenic cells had the highest level of ABA^134^. In the research of Nishiwaki et al., ABA was found to be the signal substance in the process of carrot somatic embryogenesis induced by stress^135^. In addition, ABA plays an important role in inducing the secondary embryogenesis of carrot somatic embryos^136^. Huang et al. found that genes involved in the biosynthesis and signal pathway of ABA may be affected by carotenogenesis in carrot^137^. The expression level of *DcPSY2* (phytoene synthase) can be induced by salt stress and ABA^138^. In *Arabidopsis*, salt stress was found to induce carotenoid synthesis to contribute to ABA production^139^. These results suggest a critical relationship between carotenoids and ABA.

### Cytokinins

Cytokinin (CK) is one of the five classic plant hormones and is widely used in agriculture, industry, and research^140^. Regulating cell division and differentiation is the main function of cytokinins. In some plant physiology processes, CK plays critical roles such as promoting shoot growth, inhibiting root growth, and regulating female gametophyte development^141^. CK also plays important roles in enhancing the tolerance of plants to drought, salt stress, and heat^142^. In carrots, 2-isopentenyladenine and 2-isopentenyladenosine are the major cytokinins, and 2-isopentenyladenine was only detected in carrot roots. The diameter of carrot roots is positively related to the level of CK. The concentration of endogenous CK was found to have a circadian rhythm^143^. In Chen et al., cambium tissues were found to be the cytokinin biosynthetic site in carrots. In the field production of carrots, foliar application of CK could promote the growth of carrots on nitrogen- and phosphate-depleted soil. The application of exogenous CK promotes the synthesis of endogenous CK and stimulates the growth of the cortex in carrots^144^. In the study of Wang et al., CK was thought to serve an important function in the second stage of carrot growth (40 days after sowing)^145^.

### Jasmonic acid

Jasmonic acid (JA) and its derivatives originate from lipids of chloroplast membranes^146^. This lipid-derived hormone plays critical roles in plant defense against various abiotic and biotic stresses such as pathogen infection, wounding, ultraviolet radiation, and freezing^147^. In some physiological processes of plant growth, JA also plays roles such as inhibiting root growth, and inducing leaf senescence and flowering^148^. The levels of JA in senescent leaves are significantly higher than those in nonsenescent leaves. The application of exogenous JA was reported to enhance the tolerance of *Arabidopsis* to low temperature^149^. In carrot, application of MeJA increased the total phenolics in Parano carrot. However, the concentrations of mono- and sesquiterpenes in the leaf were not influenced after MeJA treatment^150^. In Heredia and Cisneros-Zevallos’ research, MeJA application increased the accumulation of phenolics in wounded carrot tissues^151^. In the research of Wang et al., the levels of JA at different growth stages and expression profiles of genes related to JA were measured. They thought that the regulation of JA in carrots was stage-dependent and organ-specific^152^.

## Nutrition

### Carotenoids

Carotenoids are natural pigments and were found to be present in all photosynthetic organisms. Additionally, carotenoids are known to be good for human health, especially in disease prevention^9,153^. According to previous reports, carotenoids function in preventing cancer, cerebrovascular disease (CVD), human immunodeficiency virus (HIV) and cataracts. Their antioxidant properties were thought to be the main factor for the abundant functions of carotenoids^154^. In Krinsky’s research, eating fruit and vegetables that are rich in carotenoids was found to prevent many diseases, such as disorders related to the eye^155^.

In horticultural crops, studies on carotenoids have been widely performed. Tomato, pepper, and carrot were found to be carotenoid-rich vegetables^156,157^. Carrot is a kind of root vegetable that has abundant carotenoids accumulating in the root. In the research of Perrin et al., the accumulation of carotenoids was different in the carrots with different root colors. The accumulation in different root tissues was also different. In orange and purple carrot roots, the content of carotenoids in phloem was obviously higher than that in xylem. In the red genotype, the contents of carotenoids in phloem and xylem were similar. The difference in the accumulation in different root tissues was thought to be the result of the different expression patterns of carotenoid biosynthesis genes in specific tissues^158^.

The biosynthesis of carotenoids has been widely studied in plants. In 2002, Santos and Simon identified some putative QTLs that are associated with the accumulation of ξ-carotene, α-carotene, β-carotene, lycopene and phytoene in carrot^46^. In 2007, 24 potential carotenoid biosynthesis structural genes were identified by Just et al. Two phytoene synthase (*PSY)* genes (*DQ192186* and *DQ192187*) were found in carrots^159^. *PSY* is an important gene that encodes the rate-limiting enzyme in the biosynthesis pathway of carotenoids. The contents of carotenoids in the *PSY*-overexpressing lines of some vegetables were obviously enhanced^160–162^. In the sequencing of the carrot genome, a central gene (*DCAR_032551*) involved in regulating carotenoid accumulation was found. Furthermore, authors of the genome paper hypothesized that root de-etiolation may be responsible for carotenoid accumulation in carrot roots^163^. In the research of Ma et al., carotenoid contents in six different carrot cultivars were measured. They thought that carotene hydroxylase genes were involved in α-carotene accumulation and xanthophyll formation^164^. Although homologs of all known genes involved in carotenoid biosynthesis have been identified in carrots, there are still some questions that need answers, such as the accumulation of carotenoids in nonphotosynthetic carrot roots.

### Anthocyanins

Anthocyanins are widely distributed natural pigments and flavonoids^165^. Anthocyanins play critical roles in the pigmentation of flowers and fruits^166^. In many colored vegetables and fruits such as red cabbage, eggplant, purple corn, purple potato, grape, peach, plum, and pomegranate, anthocyanins were discovered^167^. In plants, anthocyanins can protect plants from strong light by forming light-absorbing screens and scavenging reactive oxygen species when suffering from light stress^168^. Anthocyanins have also been reported to have many health benefits, including prevention of cardiovascular diseases, anticarcinogenic activity, control of diabetes, and improvement of vision^165^. Providing antioxidants was determined to be the main mechanism for anthocyanins to prevent diseases in humans.

Among the cultivars of carrot, the taproot color variety is abundant. The main colors include orange, purple, yellow, red, and white. In the research of Xu et al., the taproot of purple carrots had more anthocyanin accumulation than that of yellow and orange carrots. Only a few anthocyanins were measured in the taproots of orange carrots, and no anthocyanins were measured in yellow carrot roots^169^. The accumulation of anthocyanins in the carrot roots was affected by many factors, including temperature, nutrients, and light^170^. At the molecular level, many studies on the biosynthesis pathway of anthocyanins have been performed in carrots. Phenylalanine ammonialyase (PAL), flavanone 3-hydroxylase (F3H), chalcone synthase (CHS), dihydroflavonol 4-reductase (DFR), and leucoanthocyanidin dioxygenase (LDOX) are members of the biosynthesis pathway and have been identified in carrots^171^. According to previous reports, the genes *DcUCGalT1*, *DcMYB6*, and *DcUSAGT1* of carrot were involved in the biosynthesis of anthocyanins^172–174^. In Yildiz and his group’s research, the expression profiles of six anthocyanin biosynthesis-related genes (*CHS1*, *FLS1*, *F3H*, *LDOX2*, *PAL3*, and *UFGT*) were measured. *CHS1*, *DFR2*, *F3H*, *LDOX2* and *PAL3* have the highest expression level in solid purple carrots^175^.

### Dietary fiber

Dietary fiber is a class of compounds that mainly includes carbohydrates, polysaccharides, and lignin^176,177^. The dietary fibers are further classified into soluble and insoluble fibers. Pectins, gums, and hemicelluloses are soluble fibers, and cellulose is the main component of insoluble fiber. In most foods, insoluble fiber is the main dietary fiber, and the content of soluble fibers is small or negligible. The sources of soluble fibers are limited and include citrus fruits, barley, legumes, oats, avocado, and rye. The sources of insoluble fibers are abundant and include most vegetables and cereal grains^178^.

Dietary fiber is well known and popular as a healthy substance within society. The widely known benefit of dietary fiber is its role in improving gastrointestinal function. Insoluble fiber can improve gastrointestinal function by enhancing the peristalsis of the intestine and increasing the bulk of feces^179^. Another way that insoluble fiber protects the colon is through enhancing the proliferation of microbes^178^. In addition, many other functions of dietary fiber have also been reported, such as moderating the postprandial insulin response, reducing cholesterol, regulating appetite, and reducing the risk of coronary heart disease. Sufficient intake of dietary fiber is good for humans to stay healthy^178,180^.

In the storage root of carrot, 1.2–6.44% of the mass is dietary fiber, and 80.94% of the dietary fiber is cellulose^8,181^. Carrot is a good material for producing juice, but the carrot pomace is wasted. According to previous reports, the dietary fiber of the carrot pomace was abundant and was higher than that of some other agricultural byproducts, including asparagus and onion. The insoluble dietary fiber in the carrot pomace are mainly composed of pectic polysaccharides, hemicelluloses and cellulose and have desirable functions^177^. As a byproduct, carrot pomace is very abundant and could be chosen as a source of dietary fiber. In the research of Chou et al., reducing particle sizes of the carrot insoluble fiber-rich fraction (IFF) can clearly enhance the function of fiber in gastrointestinal function. Furthermore, the ability of micronized IFF to reduce the concentrations of serum triglycerides, serum total cholesterol, and liver lipids was substantially enhanced^182^.

In the research of Wang et al., most lignin in the carrot root was deposited in the xylem. The proportions of lignin in carrot roots decreased with root development^183^. Hypoxia was also found to enhance the lignification of carrot roots^184^. As a promising source of dietary fiber, carrot should be further studied.

### Other compounds

Carrot is well known as being a good carotenoid provider. Moreover, carrot roots also contain many other beneficial contents, including vitamins, carbohydrates and minerals^6,185^. According to Li et al., sugars, glucose, fructose and starch are the main types of carbohydrates in carrot storage roots^181^. The biosynthesis of the sources is thought to be regulated by *DcSus* genes in carrot^186^. There are also many minerals in carrot roots, such as potassium, magnesium, calcium, sodium, and iron. In the research of Nicolle et al., potassium was found to be the most abundant mineral in carrots^6^. Among these minerals, the content of iron, sodium and magnesium is highly dependent on the carrot variety, whereas the content of potassium and calcium is not^187^. In addition, carrot roots are a good source of vitamin E and ascorbic acid. The concentrations of vitamin E and ascorbic acid in carrots are approximately 191−703 μg and 1.4−5.8 mg per 100 g fresh weight, respectively^6^.

## Conclusions and future perspectives

Vegetables are good sources of nutritious substances such as vitamins, minerals, and dietary fiber. With the increasing requirement for healthy diets, vegetables are becoming increasingly popular. Scientific studies on vegetables have become more necessary. Studies on vegetables have been substantially promoted by the use of many new technologies. As a nutritious root vegetable, carrots are popular and cultivated around the world. Many studies on the germplasm resource, breeding, tissue culture, and molecular research of carrots have been reported. Future research on carrots may focus on the following aspects.

### Improving the genome editing system

The CRISPR/Cas9 system is a highly efficient genome editing method developed in recent years. This system was first used in editing the human genome in 2013 and was then widely employed in editing the genomes of other species. The CRISPR/Cas9 system has been successfully applied in many plant species, including rice, *Arabidopsis thaliana*, soybean, maize, and liverwort. This system is an efficient technology for targeting and modifying DNA sequences of interest and is a useful way to study gene functions and crop improvement. However, this efficient tool has not been widely used in carrot research. Improving the CRISPR/Cas9 system in carrots and employing it for carrot improvement should be a focus in the future.

### Mining functional genes

The carrot genome has been sequenced, and the genome sequence data have been released. With the rapid development of sequencing technology, many omics technologies, such as transcriptomics, proteomics, and metabolomics, have been employed in carrot research. A large amount of data generated from these studies provides a potential reference for mining carrot functional genes. Genes controlling root genotype, disease resistance, and cytoplasmic male sterility are candidates to be further identified and determined in future research work. Mining functional genes will guide and promote the breeding work of carrots. The genes that control the swelling progress of carrot roots are still unidentified. With the development of sequencing technology, many opportunities will present themselves for research on the swelling of carrot roots.

### Developing the medicinal value of carrots

Carrot storage roots contain abundant biologically active substances. Many of these substances are important for human health. Carrots are used to make juice and dietary fiber in the food industry. In medicinal use, there has been little research on the pharmacological mechanism of many active substances in carrots. Many antioxidants, such as anthocyanins, carotenoids, and polyacetylene, provided by carrots play important roles in preventing disease. With increasing health awareness, the active substances in carrots have good research value and prospects for medicinal use. More future work may focus on developing the medicinal uses of carrots.

### Use of hormones in carrot field production

The human population is increasing rapidly around the world, and a substantial increase in agricultural productivity is needed. Through promoting plant growth and enhancing tolerance to biotic and abiotic stresses, plant hormones provide an opportunity to improve crop production. However, prohibitive costs restrict the widespread and continuous application of hormones in yield production. In carrots, there are almost no reports about the use of hormones in field production. In our opinion, plant hormone application in carrot yield production is worthy of further study. The interaction between human health and hormone application also needs to be studied. In the research on brassinosteroids, the application of brassinosteroids was reported to have no negative impact on human health. In clinical studies, only a very high concentration of brassinosteroids (1000 mg/kg) led to some undesirable side effects in rats^188,189^. However, there are few clinical studies on the application of other hormones. More future work may focus on the use of hormones in carrot field production, including application method and efficiency. At the same time, more clinical studies should be performed to investigate the interactions between human health and hormone application.
